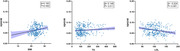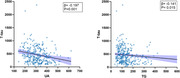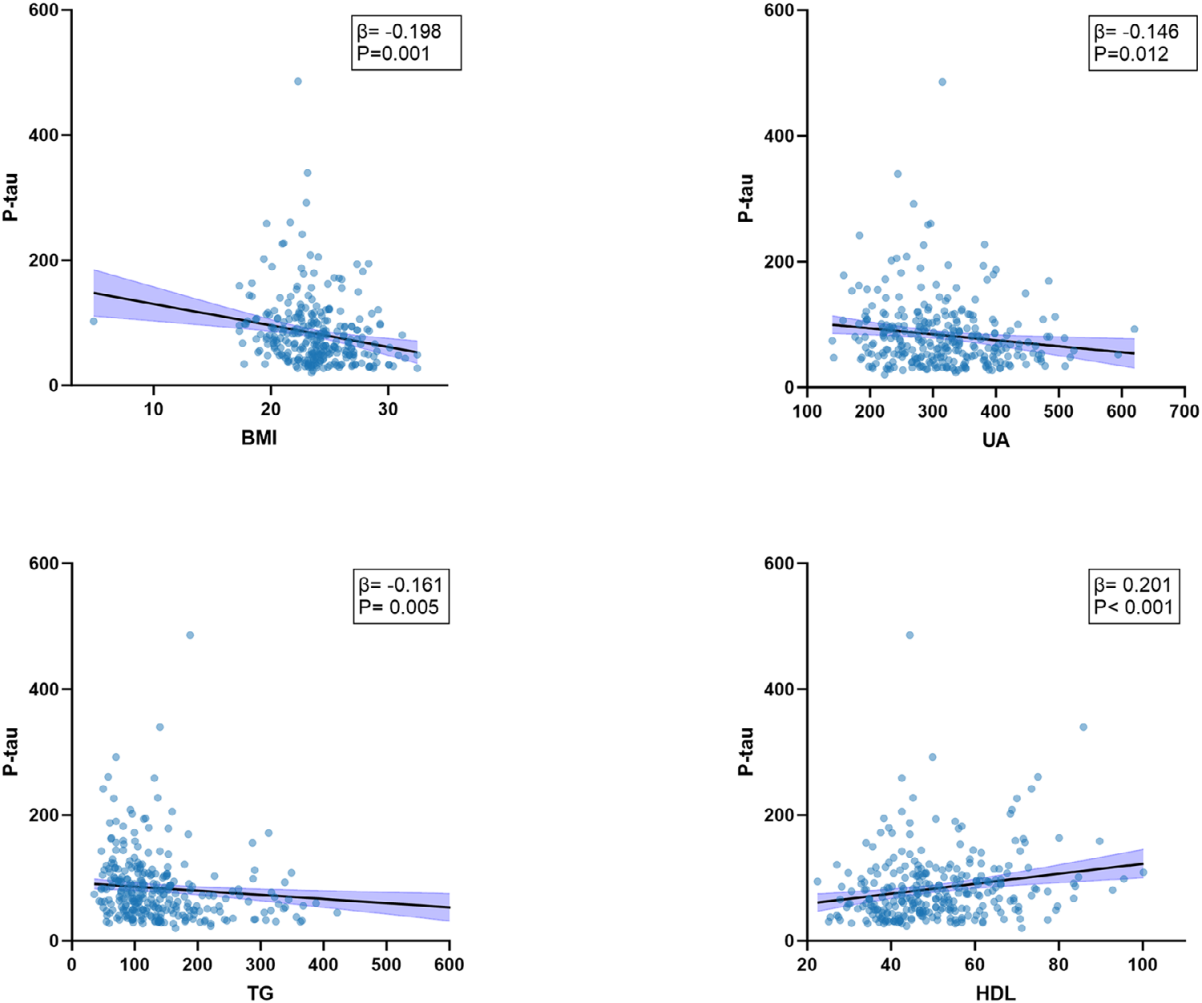# Associations between serum metabolic syndrome indicators levels and cerebrospinal fluid pathological protein

**DOI:** 10.1002/alz.088866

**Published:** 2025-01-09

**Authors:** Jiayi Ding, Meina Quan, Jianping Jia

**Affiliations:** ^1^ Innovation Center for Neurological Disorders, Xuanwu Hospital, Capital Medical University, Beijing, China;, Beijing China; ^2^ Beijing Key Laboratory of Geriatric Cognitive Disorders, Beijing China; ^3^ Key Laboratory of Neurodegenerative Diseases, Ministry of Education, Beijing China; ^4^ Center of Alzheimer’s Disease, Beijing Institute for Brain Disorders, Beijing China; ^5^ Innovation Center for Neurological Disorders, Xuanwu Hospital, Capital Medical University, Beijing, Beijing China; ^6^ National Clinical Research Center for Geriatric Disorders, Beijing China

## Abstract

**Background:**

Metabolic syndrome (MetS) was associated with an increased incidence of mild cognitive impairment (MCI) and progression to dementia. However, little is known about why this occurs. This study was to examine the correlation between the MetS indicators and cerebrospinal fluid (CSF) pathological protein biomarkers to investigate this mechanism.

**Methods:**

70 normal cognition (NC), 79 MCI, and 150 dementia participants were included, with the results of lumbar puncture and peripheral blood biochemistry. The CSF levels of amyloid (Aβ) 42, total tau (T‐Tau), phosphorylated tau (P‐Tau) and Aβ42/40 ratio were selected as the biomarkers of pathological protein. The body mass index (BMI) and the plasma high density lipoprotein cholesterol (HDL‐C), uric acid (UA), low density lipoprotein cholesterol (LDL‐C), triglyceride (TG) and homocysteine levels were selected as markers of MetS. Linear regression model was used to analyze the correlation, controlling for age, sex and apolipoprotein E genotype.

**Results:**

In all participants, BMI and TG were negatively correlated and LDL‐C was positively correlated with CSF Aβ42/40 ratio. (Figure 1) UA and TG were negatively correlated with CSF T‐tau levels.(Figure 2) BMI, UA and TG were negatively correlated and HDL‐C was positively correlated with CSF P‐Tau concentrations. (Figure 3) In the NC group, no correlation was observed. In the MCI group, LDL‐C was negatively correlated with Aβ42/40. Higher levels of UA and TG were correlated with lower CSF T‐Tau concentrations. BMI and TG levels were negatively correlated and HDL‐C was positively correlated with CSF P‐Tau levels. In the dementia group, BMI was positively associated and LDL‐C was negatively associated with CSF Aβ42/40 ratio. HDL was negatively correlated with and homocysteine was positively correlated with CSF Aβ42 levels. UA and TG were negatively correlated with CSF T‐Tau levels. TG and homocysteine levels were negatively correlated and HDL‐C was positively correlated with CSF P‐Tau levels.

**Conclusion:**

The pathological proteins concentrations in the CSF of the elderly were differentially associated with MetS indicators in different stages of dementia. These findings suggest that serum lipids modulate CSF pathological proteins, which could offer new approaches to slowing disease progress and reducing the incidence of dementia.